# The Role of Polaronic States on the Spin Dynamics in Solution‐Processed Two‐Dimensional Layered Perovskite with Different Layer Thickness

**DOI:** 10.1002/advs.202302554

**Published:** 2023-07-03

**Authors:** Mu‐Sen Song, Hai Wang, Zi‐Fan Hu, Yu‐Peng Zhang, Tian‐Yu Liu, Hai‐Yu Wang

**Affiliations:** ^1^ State Key Laboratory of Integrated Optoelectronics College of Electronic Science and Engineering Jilin University 2699 Qianjin Street Changchun 130012 China

**Keywords:** 2D perovskites, polaronic states, spin lifetime, transient absorption

## Abstract

2D lead halide perovskites (LHPs) show strong excitonic and spin‐orbit coupling effects, generating a facile spin injection. Besides, they possess a polaron character due to the soft crystal lattice, which can prolong the spin lifetime, making them favorable materials for spintronic applications. Here, the spin dynamics of 2D PEA_2_PbI_4_(MAPbI_3_)*_n_*_−l_ thin films with different layers by temperature‐ and pump fluence–dependent circularly polarization‐resolved transient absorption (TA) measurements is studied. These results indicate that the spin depolarization mechanism is gradually converted from the Maialle–Silva–Sham (MSS) mechanism to the polaronic states protection mechanism with the layer number increasing from <*n*> = 1 to 3, which is determined by the interplay between the strength of Coulomb exchange interaction and the strength of polaronic effect. While for <*n*> ≥ 4, the Elliot–Yafet (EY) impurities mechanism is proposed, in which the formed polaronic states with free charge carriers no longer play the protective role.

## Graphical abstract

The spin depolarization mechanism was gradually converted from the Maialle–Silva–Sham (MSS) mechanism to the polaronic states protection mechanism with the layer number increasing from <*n*> = 1 to 3, which is determined by the interplay between the strength of Coulomb exchange interaction and the strength of polaronic effect.

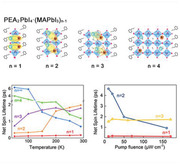

## 1 Introduction

Semiconductor spintronics making use of the electron's spin degree of freedom can pave the way for a new generation of electronic devices. The materials for spintronic devices require both efficient spin generation and a long depolarization lifetime for spin transport and detection.^[^ ^1^, ^2^ ^]^ However, these two characteristics are hard to coexist in conventional semiconductors. In general, high spin‐orbit coupling (SOC) enables efficient spin injection,^[3]^ but it typically reduces the spin lifetime.^[4]^ Therefore, suppressing spin depolarization in the presence of high SOC semiconductors is still a challenge for spintronics. Fortunately, lead‐halide perovskites (LHPs) with specific spin properties could ease this difficulty.^[^ ^5^, ^6^, ^7^ ^]^

LHPs with remarkable properties have not only unprecedentedly raised the efficiency of solar cells and the performance of luminescent devices,^[^ ^8^, ^9^, ^10^, ^11^, ^12^ ^]^ but also led to tremendous potential for spintronic devices.^[^ ^13^, ^14^, ^15^ ^]^ In particular, 2D LHPs with layer‐dependent optoelectronic characteristics and favorable stability make them a better candidate for the spintronic device.^[16]^ 2D LHPs refer to the addition of long‐chain organic groups between inorganic layers (the number of inorganic layers is expressed by *n*) on the basis of 3D perovskites, resulting in the formation of natural quantum well (QW) structures. Thus, the symmetry of the 3D system can be destroyed, and together with the high SOC in lead–iodide cages, the band edge can be strongly modified and the spin‐degeneracy can be lifted.^[17]^ Hence, compared to conventional semiconductors, the spin relaxation mechanism of 2D LHPs is a more complex subject and still far from being understood.

Generally, there are three types of spin relaxation mechanisms in conventional semiconductors known as Elliot–Yafet (EY),^[18]^ D'yakonov–Perel (DP),^[19]^ and Bir–Aronov–Pikus (BAP)^[20]^ or Maialle–Silva–Sham (MSS) mechanism (See Note S1, Supporting Information, for a more detailed description). While for 2D LHPs, spin depolarization mechanisms are affected by more factors such as sample quality, chemical composition,^[21]^ structural phases,^[22]^ exciton binding energy,^[23]^ and so on. However, to date, only a few studies explored the spin dynamics in 2D LHPs. In 2018, Chen et al. reported spin relaxation in a series of 2D LHPs single crystals with different layer thicknesses.^[24]^ A maximum spin relaxation lifetime was observed in the *n* = 4 sample, which is a balance of Rashba splitting and phonon scattering. Later, Zhu et al. reported that excitons in 2D LHP nanoplates (*n* = 3) exist as exciton polarons,^[25]^ in which the exciton–exciton interaction is weakened, resulting in a longer exciton spin lifetime at the higher temperature. It is therefore noteworthy that the formation of the polaronic state has a significant impact on spin depolarization dynamics in 2D LHPs. Indeed, due to the soft nature of the crystal, strong exciton–phonon coupling has been demonstrated in LHPs.^[^ ^26^, ^27^, ^28^ ^]^ The formed exciton–phonon coupled state is known as the polaronic state, which is considered to be a critical factor in many special properties of 2D LHPs.^[^ ^29^, ^30^, ^31^, ^32^ ^]^ However, as an essentially natural quantum wells, the structures and properties of 2D‐layered perovskites should change with the layer thickness varies, that is, the dimensional effect of the materials should play a role on their intrinsic properties. In particular, the layer thickness plays a crucial role in the determination of the exciton binding energy, which has a strong influence on the spin lifetime. But the analysis of the role of exciton binding energy and the formation of polaronic states in the spin dynamics for different layer thickness 2D perovskites are still lacking.

In this work, we investigate the spin relaxation dynamics of solution‐processed 2D PEA_2_PbI_4_(MAPbI_3_)*_n_*_−1_ film with different layer thicknesses (<*n*> = 1, 2, 3, 4, and ∞) by circularly polarization‐resolved transient absorption (TA) spectroscopy.^[33]^ Our results demonstrate that the interplay between the strength of Coulomb exchange interaction and the strength of polaronic effect contributes to the net spin relaxation. The net spin depolarization mechanism of 2D LHPs transforms gradually from the MSS mechanism to the polaronic states protection mechanism when the layer number increased from <*n*> = 1 to 3. While, for <*n*> ≥ 4 films, the initially formed free charge carriers‐polarons are easier to be scattered by the charged impurities and grain boundaries, thus belonging to the EY impurities mechanism.

## 2 Results and Discussion

**Figure 1:**
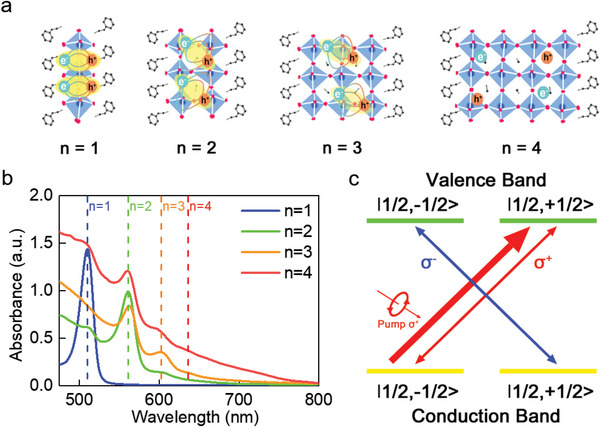
a) Crystal structures of 2D LHPs PEA_2_PbI_4_(MAPbI_3_)*_n_*_−1_ (<*n*> = 1–4). Colors: black, C; green, N; dark blue, Pb; pink, I. Inset: schematic depiction of the strength of Coulomb exchange interaction and the strength of polaronic effect. b) Absorption spectra of the 2D LHPs films at room temperature. c) Selection rule in circularly polarization‐resolved TA measurements. The total angular momentum of both the electron and hole of the band edge is *J* = 1/2, and the magnetic quantum number *m_j_* = ±1/2.

Due to the high solution processability and tolerance to defects, 2D LHPs with phenylethylamine as the organic spacers were selected since it is usually considered as the reference configuration to study the optoelectronic properties. The preparation of 2D LHPs polycrystalline thin films follows the general formula PEA_2_PbI_4_(MAPbI_3_)*_n_*_−1_, whose structures are plotted in **Figure** **1**a (<*n*> = 1, 2, 3, 4, and ∞, <*n*> represents the designed *n* value, PEA = phenylethylamine, MA = methylamine). PbI_2_, PEAI, and MAI were dissolved in the mixed solution of DMF:DMSO = 4:1 with the proportion of *n*: 2: *n* − 1. Then the precursor solution was spin‐coated on the pre‐cleaned glass substrate for 60 s under 3000 rpm and then annealed at 100 °C for 60 s in a nitrogen‐filled glove box. All chemicals are purchased from Xi'an *ρ*‐OLED. The absorption spectra of the 2D LHPs films are shown in Figure 1b. As can be seen, the <*n*> = 1 film shows a sharp peak at 511 nm since only a single phase is present. While for <*n*> ≥ 2, although the films were prepared as the general formula PEA_2_PbI_4_(MAPbI_3_)*_n_*_−1_, quasi‐2D LHPs film with mixed phases (different *n*‐values) are formed due to the formation energies of them are similar.^[34]^ Absorption peaks on behalf of <*n*> = 2, 3, 4 can be observed at 561 (<*n*> = 2), 602 (<*n*> = 3), and 637 nm (<*n*> = 4), respectively. As expected, the energy bandgap (*E*_g_) monotonically decreases with the increase of the <*n*>.

Even though multiple <*n*> value phases are created in quasi‐2D LHPs film (n ≥ 2), their intrinsic spin dynamics can be revealed by circularly polarization‐resolved TA spectroscopy under resonance excitation. For the case of nonresonant excitation, hot excitons will be generated in the low‐*n* phase, and then thermalized toward the high‐*n* phase through energy transfer. Typically, spin relaxation via momentum scattering (EY mechanism) will occur during this process. Thus, to elucidate the dominant spin relaxation mechanism in 2D LHPs film, the first series of TA measurements were performed with resonant excitation under different temperatures between 50 and 290 K. The wavelength of the pump light was selected at 515, 580, 610, and 750 nm for <*n*> = 1, 2, 3, and 4, respectively. In all the measurements, the pump light was arranged as right circular polarization (*𝜎*+), while the broadband probe light was set as right or left circular polarization (*𝜎*+/*𝜎*−) to achieve the same (SCP) or opposite (OCP) circularly polarized TA detection. Due to the conservation of angular momentum, the *σ*+ pump pulse causes the transition of the spin state |1/2, −1/2⟩ to |1/2, +1/2⟩ (the increase of angular momentum +ℏ), and each polarized probe light can detect its corresponding state (as shown in Figure 1c).

**Figure 2:**
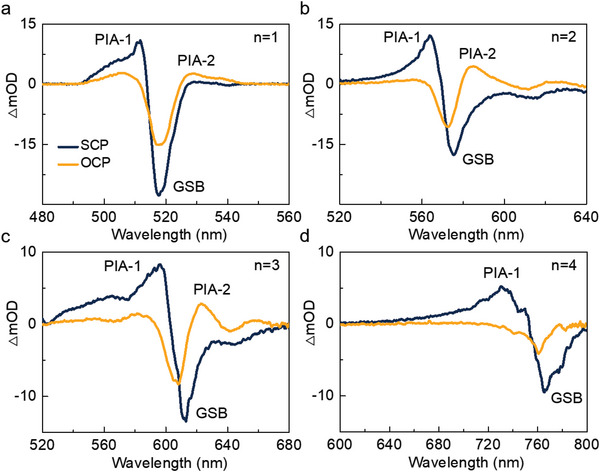
The initial SCP and OCP TA spectra at 0.4 ps of a) <*n*> = 1, b) <*n*> = 2, c) <*n*> = 3, and d) <*n*> = 4 samples at room temperature.

First, the initial SCP and OCP TA spectra of the <*n*> = 1 sample are compared in **Figure** **2**a. Under SCP configuration, the spectrum contains a ground state bleach (GSB, ΔmOD < 0) signal at 520 nm corresponding to the <*n*> = 1 exciton resonance, and a photoinduced absorption (ΔmOD > 0) at the higher energy side of the GSB signal, which has a very short lifetime arising from the Starks effect.^[35]^ While, under OCP configuration, the −1 exciton state possesses a redshift when the +1 excitons are pumped, resulting in the initial OCP TA spectra profile with a PIA2 signal. Subsequently, the −1 exciton state as the minority would receive population scatting from the +1 exciton state until the two states are balanced. Therefore, the change of the kinetics in the lower energy side of the GSB signal can be assigned as the spin polarization decay.

**Figure 3:**
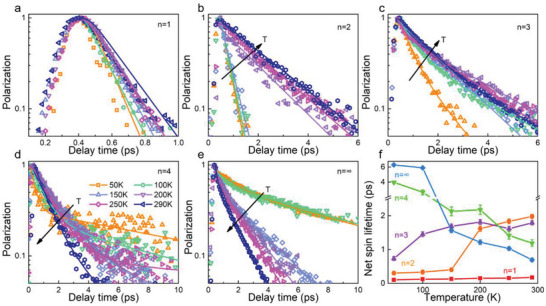
Net spin relaxation kinetics fitted with exponential decay of a) <*n*> = 1, b) <*n*> = 2, c) <*n*> = 3, d) <*n*> = 4, and e) <*n*> = ∞ samples at different temperatures. f) Net spin lifetimes versus layers of 2D LHPs films at different temperatures.

Accordingly, the net spin dynamics can be calculated from the difference between the SCP and OCP TA kinetics of the PIA2 (see Figure S1, Supporting Information). Through single‐exponential fitting, a very short net spin lifetime of ≈0.16 ps is obtained, which is consistent with previous work on <*n*> = 1 2D LPHs.^[^ ^24^, ^25^, ^26^, ^27^, ^28^, ^29^, ^30^, ^31^, ^32^, ^33^, ^34^, ^35^, ^36^ ^]^ Indeed, such fast spin depolarization is expected since the <*n*> = 1 2D LHPs possess an extremely large exciton binding energy (*E*_b_ ≈ 455 meV)^[^ ^37^ ^]^ and thus strong Coulomb exchange interaction, which is conformed to the MSS mechanism. In addition, due to the Coulomb interaction having a weak temperature dependence,^[38]^ the net spin exhibits a similar depolarization lifetime for all the temperatures as shown in **Figure** **3**f (red line).

Furthermore, with the increase of <*n*>, the spin relaxation process in quasi‐2D LHPs is affected by more factors. To clarify the dominant spin‐relaxation mechanisms in <*n*> = 2 and 3 samples, the TA spectra of the two samples are deeper analyzed. As shown in Figure 2b,c, compared with that of the <*n*> = 1 sample, the initial SCP and OCP TA spectra at 0.4 ps of the <*n*> = 2 and 3 samples display similar spectral profiles, just with peaks redshift. Hence, the spin relaxation can also be revealed by fitting the difference of the SCP and OCP kinetics at PIA2 (576 nm for <*n*> = 2 and 623 nm for <*n*> = 3 as shown in Figures S2 and S3, Supporting Information). At room temperature, nearly identical net spin lifetimes constant of 2.2 and 2.1 ps for <*n*> = 2 and 3 are obtained, which is about one order of magnitude longer than that of the <*n*> = 1 sample. Since the *E*_b_ is decreased with the layer number increases (See Note S2, Supporting Information, for details), the Coulomb exchange interaction of the excitons for <*n*> = 2 and 3 samples are weaker than that of <*n*> = 1, and therefore a slower net spin decay is expected for the samples with larger <*n*>.

Moreover, the net spin lifetime of <*n*> = 2 and 3 samples show noticeable temperature dependence, both of which are shortened with the decrease of the temperature as shown in Figure 3b,c, representing that there should be additional net spin depolarization mechanism presenting in the system. Thus, to reveal the mechanism, temperature‐dependent net spin decay time constants are extracted and plotted in Figure 3f (orange and purple line). For the <*n*> = 2 sample, we notice that the net spin decays marginally faster for temperatures ≥ 200 K. While, the net spin lifetime shows a rapid decrease from 1.6 to 0.4 ps when the temperature decreases from 200 to 150 K and then keeps at about ≈0.3–0.4 ps from 150 to 50 K. In contrast, the trend for the <*n*> = 3 sample presents a different behavior, where the net spin lifetime slightly decreases from 2 to 1.5 ps when the temperature decreases from 290 to 100 K, and then there is a sharp drop from 1.5 to 0.7 ps when the temperature further decreases to 50 K.

For traditional semiconductors, the most relevant spin‐relaxation mechanisms are DP, EY, and BAP or MSS mechanisms. The DP mechanism usually plays a major role in the system without inversion symmetry, in which spin depolarization is induced by spin‐orbit coupling (local magnetic field). Since the spin‐orbit coupling effect is nearly temperature independent, the DP mechanism would show a weakly dependent on temperature.^[21]^ In contrast, for the EY mechanism, the dominant pathway of spin depolarization arises from the momentum scattering of carriers, which always prolongs the net spin lifetime under lower temperatures.^[^ ^21^, ^39^ ^]^ Thus, we can probably rule out the DP and EY mechanism for <*n*> = 2 and 3 samples, because the net spin lifetime is shortened under lower temperatures. On the other hand, due to the strong quantum confinement of 2D LHPs, the value of *E*_b_ for <*n*> = 2 and 3 samples can be up to 275 and 188 meV, respectively.^[^ ^24^, ^25^, ^26^, ^27^, ^28^, ^29^, ^30^, ^31^, ^32^, ^33^, ^34^, ^35^, ^36^, ^37^, ^38^, ^39^, ^40^ ^]^ As previously mentioned, the MSS mechanism should still play a role in the spin‐relaxation process, similar to that in traditional semiconductor QWs or 2D transition metal dichalcogenides. But, the MSS mechanism arising from the Coulomb exchange interaction has been also suggested to have weak temperature‐dependent.^[38]^ Hence, we suggest that there should be another mechanism that leads to the unusual temperature‐dependent spin depolarization for <*n*> = 2 and 3 samples.

In fact, due to the crystal lattice being very soft, the exciton–phonon coupling is considered to be a unique factor in LHPs. Excitons in 2D perovskites are strongly influenced by surrounding lattice deformation, leading to the formation of exciton–polarons, in which the spatial separation of the polarons becomes larger (as the schematic shown in Figure 1a). These changes will reduce the Coulomb exchange interaction, which in turn allows a new mechanism that dominates exciton–polarons spin relaxation. Concretely, at elevated temperatures, the polaronic states can be formed efficiently, giving rise to a reduced overlap of the exciton wavefunctions that prolong the net spin lifetime. Whereas, the phonons will be frozen with the temperature decreasing, resulting in the exciton–phonon coupling strength decreasing and the polaronic states cannot be efficiently formed. Thus, at low temperatures, the Coulomb exchange interaction (MSS mechanism) plays the dominant role, leading to the shortened net spin lifetime, which is well consistent with the trend of the temperature‐dependent net spin lifetime of <*n*> = 2 and 3 samples as shown in Figure 3f. In addition, compared with <*n*> = 2, the *E*_b_ is further reduced in the <*n*> = 3 sample, so the net spin decay is suggested to be governed by the formation of polaronic states at much lower temperatures. This is the reason why the net spin lifetime drops slower with the temperature decreasing in the <*n*> = 3 sample.

**Figure 4:**
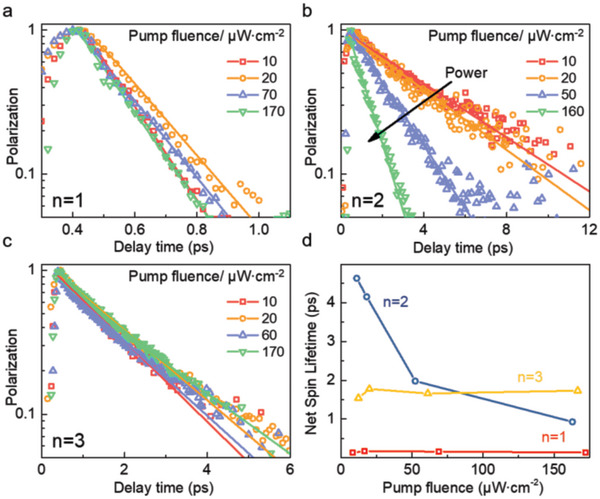
Net spin relaxation kinetics fitted with exponential decay of a) <*n*> = 1, b) <*n*> = 2, and c) <*n*> = 3 samples under different pump fluences at room temperature. d) Net spin lifetimes versus layers of 2D LHPs films under different pump fluences.

To further reveal the role of polaronic states in the net spin relaxation, we also performed pump fluence–dependent TA measurements on <*n*> = 1, 2, and 3 samples at room temperature. The net spin relaxation kinetics at different pump fluences ranging from ≈10 to ≈170 µJ cm^−2^ is obtained. First, the net spin decays for the <*n*> = 1 sample are plotted in **Figure** **4**a, which exhibits a saturated trend with increasing pump fluence. The very short net spin lifetime (≈0.16 ps, red line in Figure 4d) with weak pump fluence–dependent can still be understood by the MSS mechanism, arising from the extremely large *E*_b_ in <*n*> = 1 sample. Then, as shown in Figure 4b, with the increase of the pump fluence, the net spin lifetime for the <*n*> = 2 sample presents an obvious decrease monotonically. On the contrary, the <*n*> = 3 sample exhibits a similar decay lifetime for all the pump fluence as illustrated in Figure 4c. The fitting results of the net spin lifetime of the <*n*> = 2 and 3 are also summarized in Figure 4d. For the <*n*> = 2 sample, the net spin lifetime exhibits a strong dependence on pump fluence, in which the spin relaxation time decreases from 4.6 ps for 10 µJ cm^−2^ to 1.0 ps for 160 µJ cm^−2^. At first sight, the trend is consistent with the classical MSS mechanism, in which the Coulomb exchange interaction between excitons is enhanced with the increase of the pump fluence or the exciton densities, leading to faster spin depolarization.^[^ ^25^, ^41^ ^]^ However, different from traditional III–V group QWs and transition metal dichalcogenides, the relationship between the pump fluence and the net spin lifetime is not linear. Especially at the lowest pump fluence, a particularly long net spin lifetime up to 4.6 ps is observed. Thus, we once again propose that the formation of polaronic states has an important effect on the net spin relaxation in the <*n*> = 2 sample at low pump fluence.

In fact, it has been demonstrated that the exciton–phonon coupling can be increased by reducing the QWs width (i.e., reducing the <*n*>); while on the other side, the presence of the small polar cations between the inorganic PbI_4_^2−^ cage is also looking forward to enhancing the exciton–phonon coupling strength by coupling their dipole moment to that of the excitons.^[^ ^42^, ^43^ ^]^ Thus, bilayer (<*n*> = 2) LHP, which is the narrowest QWs containing organic cations, is considered to have the strongest exciton–phonon coupling strength. Especially for low exciton densities, the overlap of the exciton wave functions can be reduced to an extent that the Coulomb exchange interaction strength significantly drops (see <*n*> = 2 in Figure 1a). Hence, combined with the weak Coulomb exchange interaction between excitons and the more effective form of the polaronic states, an especially long net spin lifetime (4.6 ps) is observed in <*n*> = 2 sample at the low pump fluence. On the contrary, at high pump fluence, the large *E*_b_ (≈275 meV) in <*n*> = 2 sample is suggested to have a major influence on the net spin relaxation. Namely, the Coulomb exchange interaction will prevail over the effect of polaronic states at high exciton densities. Consequently, the MSS mechanism is expected to dominate the net spin relaxation, leading to the short net spin lifetime at high pump fluence.

Furthermore, for the <*n*> = 3 sample, the net spin lifetime shows a completely different picture, which is kept at about 1.7 ± 0.1 ps under different pump fluences (see Figure 4d). Different from the <*n*> = 2 sample, the *E*_b_ in the <*n*> = 3 sample is further decreased to ≈188 meV, and the Coulomb exchange interaction became weaker. Thus, we propose that the strength of polaronic effects is stronger and provides the main contribution to spin depolarization in the <*n*> = 3 sample. In other words, the exciton Coulomb exchange interaction can be largely screened even at high pump fluence in the <*n*> = 3 sample, leading to the pump fluence–independent net spin lifetime. In brief, the observed trend in Figure 4d disclosed that the net spin depolarization mechanism of 2D LHPs transform gradually from being dominated by the Coulomb exchange interaction (MSS mechanism for <*n*> = 1) to being dominated by the formation of polaronic states (<*n*> = 3). As an intermediate sample, the net spin lifetime of the <*n*> = 2 is controlled by the two counteracting factors, showing a more pronounced pump fluence–dependence.

Finally, for the <*n*> = 4 sample, the TA spectra following excitation with a 665 nm laser pulse (resonance excitation with <*n*> = 4) are shown in Figure S4, Supporting Information. As can be seen, the GSB signal of <*n*> = 4 is very weak, while the <*n*> = ∞ GSB signal has a dominant role. These features indicate that as the expected layer number <*n*> increases to 4, a broad <*n*> value was distributed in the film arising from the much lower crystallization energy barrier for the high <*n*> value.^[44]^ Even under resonance excitation with <*n*> = 4, the carriers can be fast transferred to the <*n*> = ∞ phase. Thus, the TA measurements for <*n*> = 4 and ∞ samples were carried out with a 750 nm laser pulse, and the dynamics reflecting spin difference are shown in Figures S5 and S6, Supporting Information. Accordingly, the dynamics of the net spin decay for <*n*> = 4 and <*n*> = ∞ sample show a similar temperature‐dependent characteristic as shown in Figure 3d,e. With the temperature decreasing, a significantly increased net spin lifetime can be observed as the green (<*n*> = 4) and blue (<*n*> = ∞) lines shown in Figure 3f, which show totally different trends to that have been found in <*n*> = 2 and 3 samples. Such a large contrast can be attributed to the different net spin decay mechanisms. Since the *E*_b_ falls to a much lower value (for instance, less than 139 meV in the <*n*> = 4 sample^[^ ^21^, ^24^, ^25^, ^26^, ^27^, ^28^, ^29^, ^30^, ^31^, ^32^, ^33^, ^34^, ^35^, ^36^, ^37^, ^38^, ^39^, ^40^ ^]^ and 50 meV in the <*n*> = ∞ sample^[^ ^21^, ^39^, ^40^, ^41^, ^42^, ^43^, ^44^, ^45^ ^]^), free charge carriers are initially formed. These free charge carriers‐polarons are still easier to be scattered by the charged impurities and grain boundaries. Moreover, a decreased trend of spin relaxation time with the increase of pump fluence has also been observed in MAPbI_3_ film, which reflects free charge carrier‐polarons scattering also contributes to the spin relaxation process.^[46]^ Thus, the EY impurities mechanism is generally proposed to dominate the net spin relaxation.

## 3 Conclusion

To sum up, we have studied the spin relaxation kinetics of 2D PEA_2_PbI_4_(MAPbI_3_)*_n_*_−1_ thin films by using circularly polarization‐resolved TA spectra. The effect of polaronic states on the spin relaxation mechanism for different layer thicknesses is investigated. It is found that <*n*> = 1 film has the shortest net spin lifetime, which is almost temperature‐independent. This can be attributed to the excessively strong Coulomb exchange interaction, and the spin depolarization process is even faster than the formation of polaronic states. While for the <*n*> = 2, 3 films, an unexpectedly decreased net spin lifetime is observed with the temperature reducing, which suggests that the formation of polaronic states at elevated temperatures can effectively inhibit net spin depolarization. On the contrary, the thermally activated polaronic states are no longer produced upon cooling to cryogenic temperatures, resulting in the Coulomb exchange interaction playing a leading role. Moreover, in pump fluence–dependent TA measurements, the net spin lifetime of <*n*> = 2 shows a significant pump fluence–dependent, while that of <*n*> = 3 is only slightly changed. The results further reveal that the net spin depolarization mechanism change from the MSS mechanism to the polaronic states protection mechanism with the layer number increasing from <*n*> = 1 to 3, which is determined by the interplay between the strength of Coulomb exchange interaction and the strength of polaronic effect. Finally, for <*n*> ≥ 4 films, the net spin lifetime increases with the decreasing of temperatures. In these films, the initially formed free charge carriers‐polarons are easier to be scattered by the charged impurities and grain boundaries, thus belonging to the EY impurities mechanism. Our research is not only of great value for better figuring out spin relaxation mechanisms but also opens up a new direction for spintronic devices.

## 4 Experimental Section

### Variable Temperature Circular Polarized Pump–Probe Experimental Setup

A femtosecond laser pulse with a repetition rate of 500 Hz, a central wavelength of 800 nm, pulse width of 100 fs, and pulse energy of 2 mJ was generated by a titanium‐sapphire laser amplifier (Spectra‐physics) in the pump–probe experiment. The output laser was then split into two beams, and the stronger pulse was sent to the TOPAS system to generate a pump pulse, which was then chopped to 250 Hz. The other beam, the probe beam, was produced by focusing the weaker part on a sapphire slab to generate a wide band of white light. The two pulses are then focused through convex lenses and overlapped onto the sample. The sample was placed in the variable temperature device (Advanced Research Systems). The circular polarization of the pump and probe pulses were controlled by two sets of wideband Glan Polarizers and quarter‐wave plates (310–1100 nm, Thorlabs). The time delay between the pump and probe pulse was controlled by a mechanical delay line stage (DL325, Newport). Finally, the differential transmission probe signal was recorded by a spectrometer (AvaSpec‐ULS2048CL‐EVO Avantes). A chirp program was used to compensate the group velocity dispersion of the TA spectra.

## Conflict of Interest

The authors declare no conflict of interest.

## Supporting information

Supporting Information

## Data Availability

Research data are not shared.

## References

[1] O. Z. Karimov , G. H. John , R. T. Harley , W. H. Lau , M. E. Flatte , M. Henini , R. Airey , Phys. Rev. Lett. 2003, 91, 246601. 14683140 10.1103/PhysRevLett.91.246601

[2] K. C. Hall , W. H. Lau , K. Gündoğdu , M. E. Flatté , T. F. Boggess , Appl. Phys. Lett. 2003, 83, 2937.

[3] J. C. Sanchez , L. Vila , G. Desfonds , S. Gambarelli , J. P. Attane , J. M. De Teresa , C. Magen , A. Fert , Nat. Commun. 2013, 4, 2944. 24343336 10.1038/ncomms3944

[4] A. Manchon , H. C. Koo , J. Nitta , S. M. Frolov , R. A. Duine , Nat. Mater. 2015, 14, 871. 26288976 10.1038/nmat4360

[5] J. Wang , C. Zhang , H. Liu , R. McLaughlin , Y. Zhai , S. R. Vardeny , X. Liu , S. McGill , D. Semenov , H. Guo , R. Tsuchikawa , V. V. Deshpande , D. Sun , Z. V. Vardeny , Nat. Commun. 2019, 10, 129. 30631053 10.1038/s41467-018-07952-x PMC6328620

[6] C. Zhang , D. Sun , C. X. Sheng , Y. X. Zhai , K. Mielczarek , A. Zakhidov , Z. V. Vardeny , Nat. Phys. 2015, 11, 427. 10.1103/PhysRevLett.114.116601 25839297

[7] H. Lu , J. Wang , C. Xiao , X. Pan , X. Chen , R. Brunecky , J. J. Berry , K. Zhu , M. C. Beard , Z. V. Vardeny , Sci. Adv. 2019, 5, eaay0571. 31840072 10.1126/sciadv.aay0571 PMC6897542

[8] J. Pan , S. P. Sarmah , B. Murali , I. Dursun , W. Peng , M. R. Parida , J. Liu , L. Sinatra , N. Alyami , C. Zhao , E. Alarousu , T. K. Ng , B. S. Ooi , O. M. Bakr , O. F. Mohammed , J. Phys. Chem. Lett. 2015, 6, 5027. 26624490 10.1021/acs.jpclett.5b02460

[9] M. A. Becker , R. Vaxenburg , G. Nedelcu , P. C. Sercel , A. Shabaev , M. J. Mehl , J. G. Michopoulos , S. G. Lambrakos , N. Bernstein , J. L. Lyons , T. Stoferle , R. F. Mahrt , M. V. Kovalenko , D. J. Norris , G. Raino , A. L. Efros , Nature 2018, 553, 189. 29323292 10.1038/nature25147

[10] Y. Cao , N. Wang , H. Tian , J. Guo , Y. Wei , H. Chen , Y. Miao , W. Zou , K. Pan , Y. He , H. Cao , Y. Ke , M. Xu , Y. Wang , M. Yang , K. Du , Z. Fu , D. Kong , D. Dai , Y. Jin , G. Li , H. Li , Q. Peng , J. Wang , W. Huang , Nature 2018, 562, 249. 30305742 10.1038/s41586-018-0576-2

[11] H. Zhou , Q. Chen , G. Li , S. Luo , T. B. Song , H. S. Duan , Z. Hong , J. You , Y. Liu , Y. Yang , Science 2014, 345, 542. 25082698 10.1126/science.1254050

[12] C. He , X. Liu , Light: Sci. Appl. 2023, 12, 15. 36641482 10.1038/s41377-022-01010-4 PMC9840605

[13] Y. Ping , J. Z. Zhang , J. Phys. Chem. Lett. 2018, 9, 6103. 30281312 10.1021/acs.jpclett.8b02498

[14] M. Kepenekian , J. Even , J. Phys. Chem. Lett. 2017, 8, 3362. 28661150 10.1021/acs.jpclett.7b01015

[15] C. Li , X. Wang , Y. Wu , F. Liang , F. Wang , X. Zhao , H. Yu , H. Zhang , Light: Sci. Appl. 2020, 9, 193. 33298831 10.1038/s41377-020-00427-z PMC7687908

[16] G. Na , L. Zhang , Light: Sci. Appl. 2020, 9, 106. 32577222 10.1038/s41377-020-0340-x PMC7305146

[17] C. C. Stoumpos , D. H. Cao , D. J. Clark , J. Young , J. M. Rondinelli , J. I. Jang , J. T. Hupp , M. G. Kanatzidis , Chem. Mater. 2016, 28, 2852.

[18] R. J. Elliott , Phys. Rev. 1954, 96, 266.

[19] M. Dyakonov , Soviet Physics ‐ JETP 1971, 60, 1053.

[20] G. Bir , A. Aronov , G. Pikus , Soviet Physics ‐ JETP 1975, 42, 705.

[21] M. Zhou , J. S. Sarmiento , C. Fei , X. Zhang , H. Wang , J. Phys. Chem. Lett. 2020, 11, 1502. 32017571 10.1021/acs.jpclett.0c00004

[22] Y. Liu , H. Lu , J. Niu , H. Zhang , S. Lou , C. Gao , Y. Zhan , X. Zhang , Q. Jin , L. Zheng , AIP Adv. 2018, 8, 10.1063/1.5042489

[23] X. Chen , H. Lu , K. Wang , Y. Zhai , V. Lunin , P. C. Sercel , M. C. Beard , J. Am. Chem. Soc. 2021, 143, 19438. 34767709 10.1021/jacs.1c08514

[24] X. Chen , H. Lu , Z. Li , Y. Zhai , P. F. Ndione , J. J. Berry , K. Zhu , Y. Yang , M. C. Beard , ACS Energy Lett. 2018, 3, 2273.

[25] W. Tao , Q. Zhou , H. Zhu , Sci. Adv. 2020, 6, eabb7132. 33219022 10.1126/sciadv.abb7132 PMC7679171

[26] E. Cinquanta , D. Meggiolaro , S. G. Motti , M. Gandini , M. J. P. Alcocer , Q. A. Akkerman , C. Vozzi , L. Manna , F. De Angelis , A. Petrozza , S. Stagira , Phys. Rev. Lett. 2019, 122, 166601. 31075027 10.1103/PhysRevLett.122.166601

[27] S. A. Bretschneider , I. Ivanov , H. I. Wang , K. Miyata , X. Zhu , M. Bonn , Adv. Mater. 2018, 30, 10.1002/adma.201707312 29847699

[28] Z. Li , Y. Yan , M. S. Song , J. Y. Xin , H. Y. Wang , H. Wang , Y. Wang , J. Phys. Chem. Lett. 2022, 13, 4073. 35499477 10.1021/acs.jpclett.2c00698

[29] F. Thouin , A. R. S. Kandada , D. A. Valverde‐Chávez , D. Cortecchia , I. Bargigia , A. Petrozza , X. Yang , E. R. Bittner , C. Silva , Chem. Mater. 2019, 31, 7085.

[30] F. Thouin , D. A. Valverde‐Chavez , C. Quarti , D. Cortecchia , I. Bargigia , D. Beljonne , A. Petrozza , C. Silva , A. R. S. Kandada , Nat. Mater. 2019, 18, 349. 30643234 10.1038/s41563-018-0262-7

[31] H. G. Duan , V. Tiwari , A. Jha , G. R. Berdiyorov , A. Akimov , O. Vendrell , P. K. Nayak , H. J. Snaith , M. Thorwart , Z. Li , M. E. Madjet , R. J. D. Miller , J. Am. Chem. Soc. 2020, 142, 16569. 32869985 10.1021/jacs.0c03970 PMC7586332

[32] J. M. Urban , G. Chehade , M. Dyksik , M. Menahem , A. Surrente , G. Trippe‐Allard , D. K. Maude , D. Garrot , O. Yaffe , E. Deleporte , P. Plochocka , M. Baranowski , J. Phys. Chem. Lett. 2020, 11, 5830. 32597181 10.1021/acs.jpclett.0c01714

[33] L. Y. Zhao , H. Wang , H. Y. Wang , Q. Zhou , X. L. Zhang , T. Cui , L. Wang , T. Y. Liu , Y. X. Han , Y. Luo , Y. Y. Yue , M. S. Song , H. B. Sun , PhotoniX 2022, 3, 5.

[34] L. N. Quan , M. Yuan , R. Comin , O. Voznyy , E. M. Beauregard , S. Hoogland , A. Buin , A. R. Kirmani , K. Zhao , A. Amassian , D. H. Kim , E. H. Sargent , J. Am. Chem. Soc. 2016, 138, 2649. 26841130 10.1021/jacs.5b11740

[35] T. Y. Liu , H. Wang , M. S. Song , L. Y. Zhao , Z. F. Hu , H. Y. Wang , Laser Photonics Rev. 2022, 16, 2200176.

[36] D. Giovanni , W. K. Chong , Y. Y. F. Liu , H. A. Dewi , T. Yin , Y. Lekina , Z. X. Shen , N. Mathews , C. K. Gan , T. C. Sum , Adv. Sci. 2018, 5, 1800664. 10.1002/advs.201800664 PMC6193146 30356921

[37] M. Righetto , D. Giovanni , S. S. Lim , T. C. Sum , Appl. Phys. Rev. 2021, 8, 011318.

[38] C. R. Zhu , K. Zhang , M. Glazov , B. Urbaszek , T. Amand , Z. W. Ji , B. L. Liu , X. Marie , Phys. Rev. B 2014, 90, 10.1103/PhysRevB.90.161302

[39] A. D. Wright , C. Verdi , R. L. Milot , G. E. Eperon , M. A. Pérez‐Osorio , H. J. Snaith , F. Giustino , M. B. Johnston , L. M. Herz , Nat. Commun. 2016, 7, 11755. 10.1038/ncomms11755 PMC4894981 27225329

[40] J. C. Blancon , A. V. Stier , H. Tsai , W. Nie , C. C. Stoumpos , B. Traoré , L. Pedesseau , M. Kepenekian , F. Katsutani , G. T. Noe , J. Kono , S. Tretiak , S. A. Crooker , C. Katan , M. G. Kanatzidis , J. J. Crochet , J. Even , A. D. Mohite , Nat. Commun. 2018, 9, 2254. 29884900 10.1038/s41467-018-04659-x PMC5993799

[41] F. Mahmood , Z. Alpichshev , Y. H. Lee , J. Kong , N. Gedik , Nano Lett. 2018, 18, 223. 29239177 10.1021/acs.nanolett.7b03953

[42] H. Long , X. Peng , J. Lu , K. Lin , L. Xie , B. Zhang , L. Ying , Z. Wei , Nanoscale 2019, 11, 21867. 31696891 10.1039/c9nr06834a

[43] S. Wang , J. Ma , W. Li , J. Wang , H. Wang , H. Shen , J. Li , J. Wang , H. Luo , D. Li , J. Phys. Chem. Lett. 2019, 10, 2546. 31050442 10.1021/acs.jpclett.9b01011

[44] X. Lv , X. M. Dong , Z. Ye , J. S. Zhou , F. Deng , Y. Z. Zheng , X. Tao , Sol. RRL 2019, 3, 1800313.

[45] D. M. Niedzwiedzki , H. Zhou , P. Biswas , J. Phys. Chem. C 2022, 126, 1046.

[46] D. Giovanni , H. Ma , J. Chua , M. Grätzel , R. Ramesh , S. Mhaisalkar , N. Mathews , T. C. Sum , Nano Lett. 2015, 15, 1553. 25646561 10.1021/nl5039314

