# Supplementary material for: The Role of Polaronic States on the Spin Dynamics in Solution‐Processed Two‐Dimensional Layered Perovskite with Different Layer Thickness
Source: Adv Sci (Weinh). 2023 Jul 3;10(26):2302554. doi: 10.1002/advs.202302554 (PMC10502664; doi:10.1002/advs.202302554)
Supplement: Supplementary file 1 — Supporting Information [file ADVS-10-2302554-s001.pdf]

ADVANCED SCIENCE
Open Access

Supporting Information

for *Adv. Sci.*, DOI 10.1002/advs.202302554

The Role of Polaronic States on the Spin Dynamics in Solution-Processed Two-Dimensional Layered Perovskite with Different Layer Thickness

Mu-Sen Song, Hai Wang, Zi-Fan Hu, Yu-Peng Zhang, Tian-Yu Liu and Hai-Yu Wang**

The Role of Polaronic States on the Spin Dynamics in Solution-Processed Two-Dimensional Layered Perovskite with Different Layer Thickness

Mu-Sen Song, Hai Wang, Zi-Fan Hu, Yu-Peng Zhang, Tian-Yu Liu and Hai-Yu Wang**

State Key Laboratory of Integrated Optoelectronics, College of Electronic Science and Engineering, Jilin University

Supplementary Note 1:

1. Elliot-Yafet spin depolarization mechanism

Spin relaxation is caused by momentum relaxation in the Elliot-Yafet (EY) mechanism, in which the spin depolarization rate for III-V semiconductors can be written as:

$$\frac{1}{\tau_s^{EY}} = A\left(\frac{k_B \mathrm{T}}{E_g}\right)^2 \eta^2 \left(\frac{1-\frac{\eta}{2}}{1-\frac{\eta}{3}}\right)^2 \frac{1}{\tau_p} \tag{1}$$

where E_g is the band gap, $\tau_s{}^{\mathrm{EY}}$ is the spin lifetime in the EY mechanism, τ_p is the momentum relaxation time, A is a dimensionless constant, and $\eta = \frac{\Delta}{\mathrm{E}_g + \Delta}$, Δ is the spin-orbit split of the valence band. It can be seen from the above formula $\tau_s{}^{\mathrm{EY}} \propto \frac{1}{T^2}$, $\tau_s{}^{\mathrm{EY}} \propto \tau_p$. When the temperature is reduced, the momentum relaxation slows down because the phonon scattering becomes weaker (τ_p increase). Overall, $\tau_s{}^{\mathrm{EY}}$ should increase when the temperature is reduced in the EY mechanism.

2. D'yakonov-Perel spin depolarization mechanism

The D'yakonov-Perel (DP) mechanism is derived from the spin-orbit splitting in semiconductors with non-central inverse symmetry. In the DP mechanism for III-V semiconductors, the spin depolarization rate can be written as:

$$\frac{1}{\tau_s^{DP}} = Q\alpha^2 \frac{(k_B T)^3}{\hbar^2 E_g} \tag{2}$$

where Q is a dimensionless constant, $\tau_s{}^{\mathrm{DP}}$ is the spin lifetime in the DP mechanism and α is given by:

$$\alpha = \frac{4\eta}{\sqrt{3-\eta}} \frac{m_c}{m_0} \tag{3}$$

where m_c is the effective mass of the conduction electrons and m_0 is the effective mass of the electron rest mass. $\tau_s{}^{DP}$ is not only related to T, but also closely related to τ_p.

3. Bir−Aronov−Pikus mechanism

The Bir−Aronov−Pikus (BAP) mechanism is due to the long-range exchange interaction between an electron and a hole. However, the BAP mechanism focuses on the case of doped samples, where excitonic states are unlikely to form due to the strong screening by the majority carrier population. Therefore, the BAP mechanism describes the spin flip of a free electron upon scattering with a hole via the long-range exchange interaction.

4. Maialle-Silva-Sham mechanism

In the QWs system, the spin-flip arising from the Coulomb exchange interaction is known as Maialle-Silva-Sham (MSS) mechanism, which is more suitable for 2D LHPs with QWs.

According to the MSS mechanism, the spin depolarization rate can be expressed as:

$$1/\tau_s = \langle \Omega_K^2 \rangle \tau_p \tag{4}$$

where $\langle \Omega_K^2 \rangle$ is the square of the magnetic field average over exciton states and τ_p is the exciton scattering time.[1] The effective magnetic field Ω_K is proportional to the wave vector of the exciton (K) and the electron−hole exchange strength J. Because J has been generally assumed to be temperature (T)-independent, the thermal distribution of K yields the T dependence of Ω_K in the MSS model. As T increases, exciton distribution in K space is governed by thermal broadening, and average over thermal distribution yields a linear dependence of $\langle \Omega_K^2 \rangle$ on T.[2,3] The phonon scattering can be roughly estimated from the PL line width (Γ) by $\tau_p \propto 1/\Gamma$. Γ can be expressed as following phenomenological equation:

$$\Gamma(T) = \Gamma_0 + A/\left(e^{E_{ph}/k_B T} - 1\right) \tag{5}$$

where Γ_0 is the T-independent contribution, E_{ph} is effective phonon energy and a constant A.[4,5]

With the above analysis, it can be seen that τ_s is influenced by the two factors: $\langle \Omega_K^2 \rangle$ increases as T increase, but τ_p decreases as T increase. Nevertheless, with the temperature decreasing, a weakly increasing trend of τ_s can be modeled from equation

4,[6] which is similar to the temperature dependence for exciton spin relaxation in transition metal dichalcogenides (TMDCs) under the classical MSS mechanism.[7-9]

Supplementary Note 2:

According to J. C. Blancon's work[10], a quantitative estimate of the exciton binding energy can be obtained by the following equation:

$$E_b = E_0 \big/ \left(1+(\alpha-3)/2\right)^2 \tag{6}$$

$$\alpha = 3-\gamma e^{-L_W/2a_0} \tag{7}$$

where E_0 is 3D Rydberg energy of 3D perovskites, a_0 is Bohr radius of 3D perovskites, and L_w is physical width of the quantum well (≈ 0.63×n, 0.63 nm is only one PbI_4^{2-} octahedral physical width). The factor γ is empirically account for the deviations, including electron or hole densities and dielectric confinement effects. In certain perovskite materials, it can be treated as a constant. This finding explains the decrease in exciton binding energy as the layers increase. Here E_0 = 16 meV and a_0 = 4.6 nm.[11] We assumed γ = 1.74 to estimate the exciton binding energies of different <n> values. And then we get E_{b1} ≈ 455 meV, E_{b2} ≈ 275 meV, E_{b3} ≈ 188 meV and E_{b4} ≈ 139 meV. The estimated exciton binding energies are consistent with the results of other works.[12-16]

Supplementary Figures:

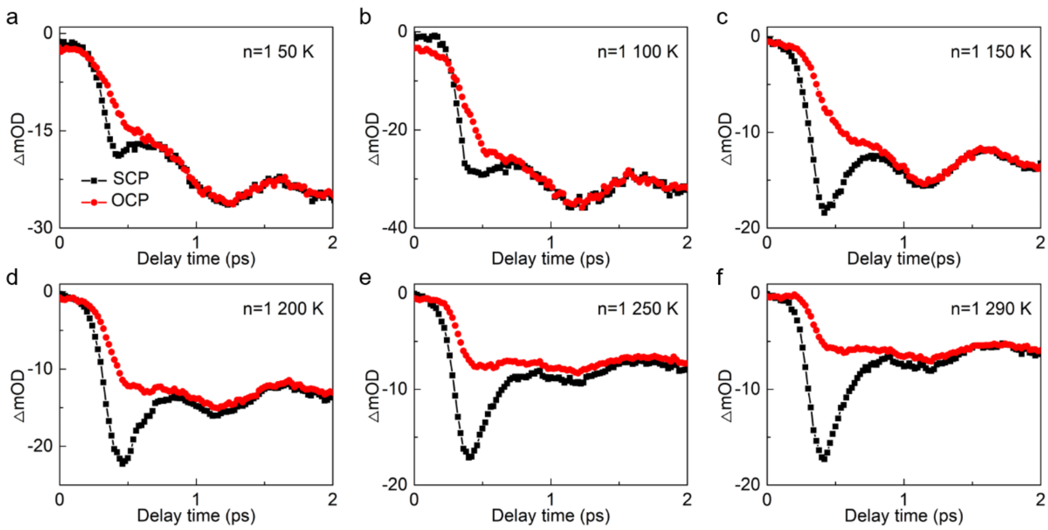

Figure S1. (a-f) The circularly polarized exciton kinetics of <n> = 1 under 515 nm laser pulse resonance excitation at 50 K, 100 K, 150 K, 200 K, 250 K, and 290 K respectively.

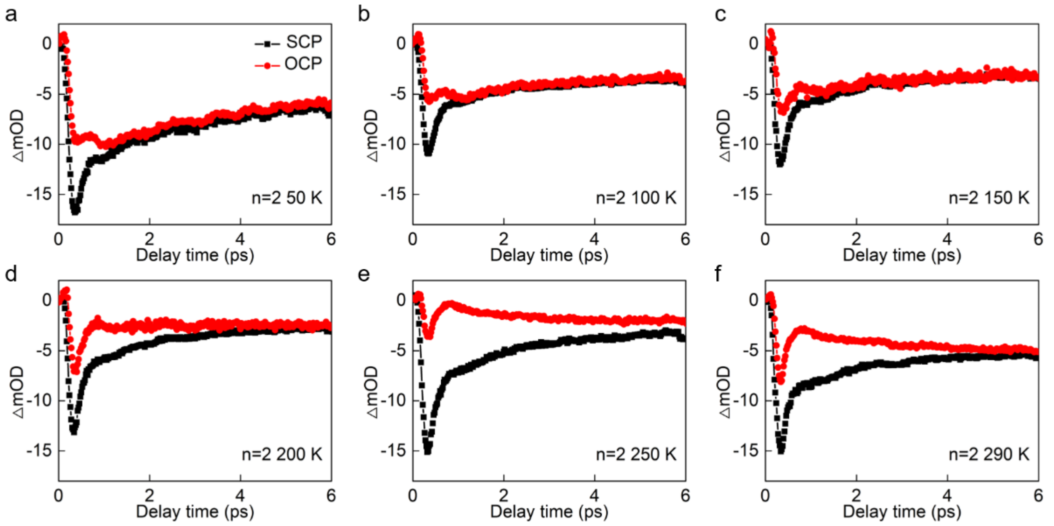

Figure S2. (a-f) The circularly polarized exciton kinetics of <n> = 2 under 580 nm laser pulse resonance excitation at 50 K, 100 K, 150 K, 200 K, 250 K, and 290 K respectively.

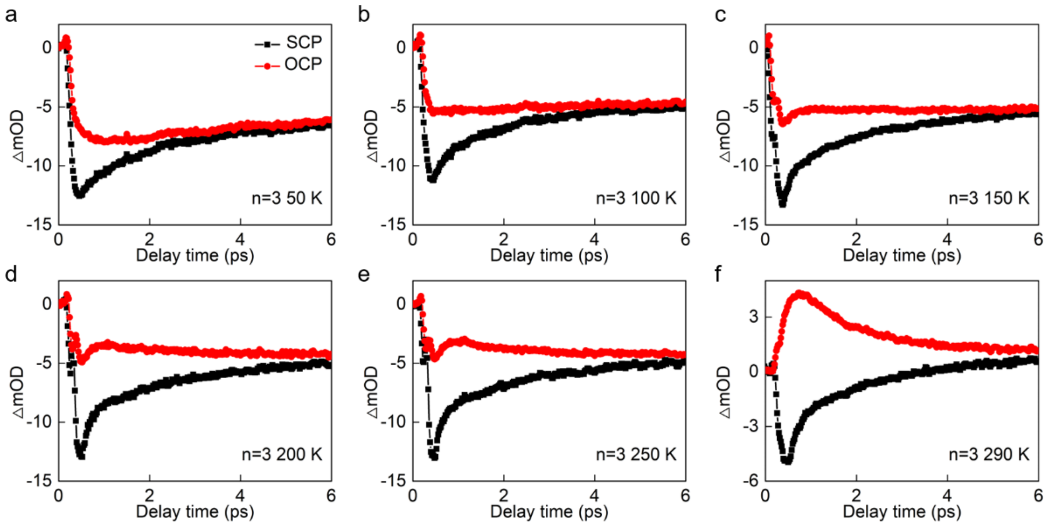

Figure S3. (a-f) The circularly polarized exciton kinetics of <n> = 3 under 610 nm laser pulse resonance excitation at 50 K, 100 K, 150 K, 200 K, 250 K, and 290 K respectively.

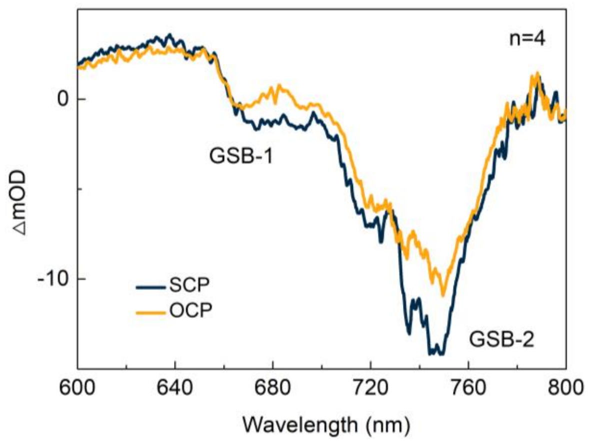

Figure S4. The circularly polarized TA spectra of <n> = 4 under 665 nm laser pulse resonance excitation.

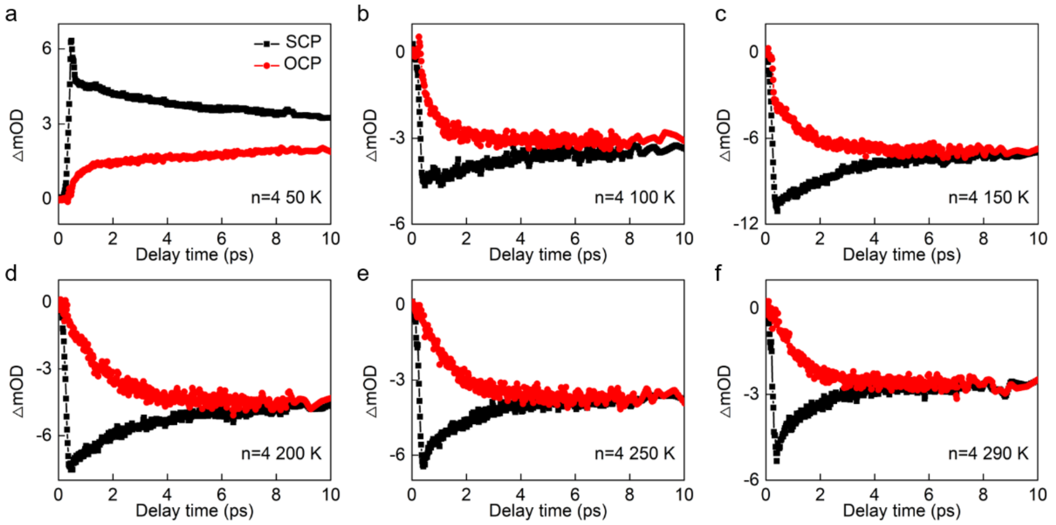

Figure S5. (a-f) The circularly polarized free charge carrier kinetics of <n> = 4 under 750 nm laser pulse excitation at 50 K, 100 K, 150 K, 200 K, 250 K, and 290 K respectively.

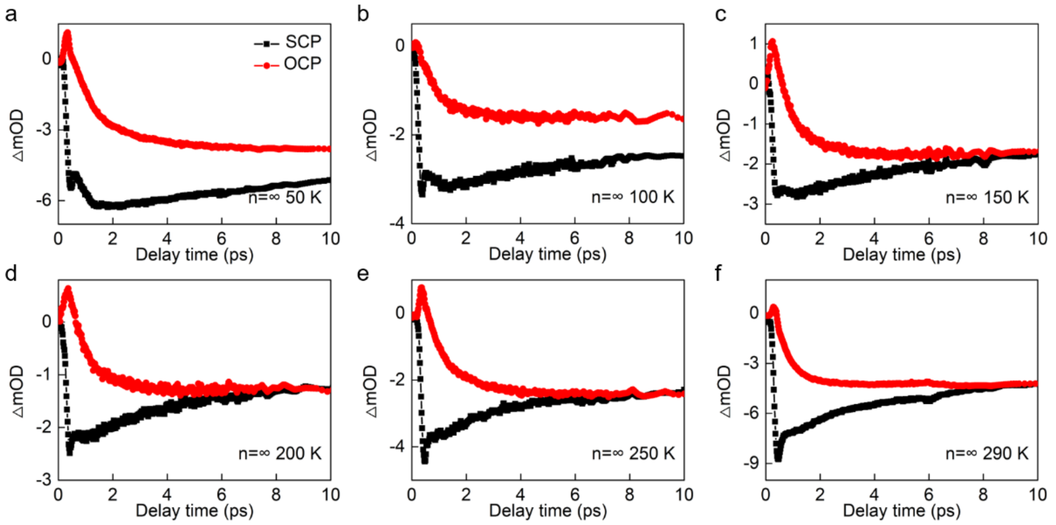

Figure S6. (a-f) The circularly polarized free charge carrier kinetics of <n> = ∞ under 750 nm laser pulse excitation at 50 K, 100 K, 150 K, 200 K, 250 K, and 290 K respectively.

Reference:

[1] M. Z. Maialle, E. A. de Andrada e Silva, L. J. Sham. *Phys. Rev. B* **1993** *47*, 15776-15788.

[2] F. Mahmood, Z. Alpichshev, Y. H. Lee, J. Kong, N. Gedik. *Nano Lett.* **2018** *18*, 223-228.

[3] L. Muñoz, E. Pérez, L. Viña, K. Ploog. *Phys. Rev. B* **1995** *51*, 4247-4257.

[4] Y. Guo, O. Yaffe, T. D. Hull, J. S. Owen, D. R. Reichman, L. E. Brus. *Nat. Commun.* **2019** *10*, 1175.

[5] Z. Guo, X. Wu, T. Zhu, X. Zhu, L. Huang. *ACS Nano.* **2016** *10*, 9992-9998.

[6] W. Tao, Q. Zhou, H. Zhu. *Sci. Adv.* **2020**, *6*, eabb7132.

[7] C. R. Zhu, K. Zhang, M. Glazov, B. Urbaszek, T. Amand, Z. W. Ji, B. L. Liu, X. Marie. *Phys. Rev. B* **2014**, *90*, 161302.

[8] F. Mahmood, Z. Alpichshev, Y.-H. Lee, J. Kong, N. Gedik. *Nano Lett.* **2018**, *18*, 223-228.

[9] S. Dal Conte, F. Bottegoni, E. A. A. Pogna, D. De Fazio, S. Ambrogio, I. Bargigia, C. D'Andrea, A. Lombardo, M. Bruna, F. Ciccacci, A. C. Ferrari, G. Cerullo, M. Finazzi. *Phys. Rev. B* **2015**, *92*, 235425.

[10] J. C. Blancon, A. V. Stier, H. Tsai, W. Nie, C. C. Stoumpos, B. Traoré, L. Pedesseau, M. Kepenekian, F. Katsutani, G. T. Noe, J. Kono, S. Tretiak, S. A. Crooker, C. Katan, M. G. Kanatzidis, J. J. Crochet, J. Even, A. D. Mohite. *Nat. Commun.* **2018**, *9*, 2254.

[11] Z. Jiang, Z. Liu, Y. Li, W. Duan. *Phys. Rev. Lett.* **2017**, *118*, 266401.

[12] X. Chen, H. Lu, Z. Li, Y. Zhai, P. F. Ndione, J. J. Berry, K. Zhu, Y. Yang, M. C. Beard. *ACS Energy Lett.* **2018**, *3*, 2273-2279.

[13] M. Righetto, D. Giovanni, S. S. Lim, T. C. Sum. *Appl. Phys. Rev.* **2021**, *8*.

[14] M. Zhou, J. S. Sarmiento, C. Fei, X. Zhang, H. Wang. *J. Phys. Chem. Lett.* **2020**, *11*, 1502-1507.

[15] J. C. Blancon, A. V. Stier, H. Tsai, W. Nie, C. C. Stoumpos, B. Traoré, L. Pedesseau, M. Kepenekian, F. Katsutani, G. T. Noe, J. Kono, S. Tretiak, S. A. Crooker, C. Katan, M. G. Kanatzidis, J. J. Crochet, J. Even, A. D. Mohite. *Nat. Commun.* **2018**, *9*, 2254.

[16] D. M. Niedzwiedzki, H. Zhou, P. Biswas. *J. Phys. Chem. C* **2022**, *126*, 1046-1054.
